# Case report: Interdisciplinary treatment of complex C1/C2 fractures in a patient with concomitant three-vessel coronary artery disease requiring bypass surgery

**DOI:** 10.3389/fsurg.2023.1123947

**Published:** 2023-05-16

**Authors:** M. E. Di Francesco, H. Magunia, A. Örgel, M. Tatagiba, M. Radwan, S. D. Adib

**Affiliations:** ^1^ Department of Neurosurgery, University of Tuebingen, Tuebingen, Germany; ^2^ Department of Anesthesiology and Intensive Care Medicine, University of Tuebingen, Tuebingen, Germany; ^3^ Department of Diagnostic and Interventional Neuroradiology, University of Tuebingen, Tuebingen, Germany; ^4^ Department of Thoracic and Cardiovascular Surgery, University of Tuebingen, Tuebingen, Germany

**Keywords:** Jefferson's fracture, odontoid fracture, coronary artery disease, intradiploic arachnoid cyst, interdisciplinary

## Abstract

**Background:**

Acute myocardial infarction (MI) frequently leads to consciousness disturbance following hemodynamic collapse. Therefore, MI can occur together with upper cervical spine trauma. Herein, we report the successful treatment of complex C1/C2 fractures in a patient with concomitant three-vessel coronary artery disease (CAD).

**Case presentation:**

A 70-year-old patient presented in our emergency outpatient clinic after a hemodynamic collapse without neurological deficits or heart-related complaints. Computed tomography (CT) scan of the cervical spine revealed a dislocated odontoid fracture Anderson and D'Alonzo type II and an unstable Gehweiler type III injury (Jefferson's fracture). An intradiploic arachnoid cyst in the posterior wall of the posterior fossa was a coincident radiological finding. Furthermore, coronary angiography confirmed three-vessel CAD with high-grade coronary artery stenosis. Indication for upper cervical spine surgery and bypass surgery was given. An interdisciplinary team of neurosurgeons, cardiothoracic surgeons and anesthesiologists evaluated the patient's case to develop the most suitable therapy concept and alternative strategies. Finally, in first step, C1-C2 fusion was performed by Harms technique under general anesthesia with x-ray guidance, spinal neuronavigation, Doppler ultrasound and cardiopulmonary monitoring. Cardiothoracic surgeons were on standby. One month later bypass surgery was performed uneventfully. Follow-up CT scan of cervical spine revealed intraosseous screw positioning and beginning fusion of the fractures. The patient did not develop neurological deficits and recovered completely from both surgeries.

**Conclusions:**

Treating complex C1/C2 fractures with concomitant severe CAD requiring treatment is challenging and carries a high risk of complications. To our knowledge, the literature does not provide any guidelines regarding therapy of this constellation. To receive upper cervical spine stability and to prevent both, spinal cord injury and cardiovascular complications, an individual approach is required. Interdisciplinary cooperation to determine optimal therapeutic algorithms is needed.

## Introduction

Injuries to the cervical spine occur in 2%–4% of all patients on admission to the emergency room, but may be life-threatening if associated with spinal cord injury (SCI) (1). Between 19%–51% of spinal trauma cases involve injuries to the cervical spine (2), and approximately 30% affecting the atlantoaxial region, with the odontoid being the most common site of injury to the C2 vertebra (3–6). Typical causes of accidents are high-velocity impact in polytraumatized patients and increasingly low-energy trauma e.g., domestic collapses in the elderly due to hyperextension on head impact (7, 8). Identification of craniovertebral junction (CVJ) injury is difficult as patients often present with reduced level of consciousness. Related lesions are traumatic brain injury, additional fractures of the spine, the midface, the extremities (2, 9) and vertebral artery injury (10–12). Hence, immediate neuroradiological diagnostic is crucial, dictating patient management.

One of the most common reasons for emergency department admission remains chest pain caused by acute coronary syndrome. Myocardial infarction (MI) often manifests itself atypically e.g., dyspnea, cardiac arrhythmias, abdominal pain and autonomic dysregulation. The last is observed especially in women, the elderly and patients with diabetes mellitus or chronic renal insufficiency (13, 14). In addition, acute MI can lead to syncope, consciousness disturbance and cerebral dysfunction following hemodynamic collapse (15). Therefore, upper cervical spine trauma can occur together with MI (16). Treating complex CVJ-fractures with coronary artery disease (CAD) requiring treatment is challenging and carries a high risk of complications. To our knowledge, the literature does not provide any guidelines regarding the therapy of this constellation. Herein, we report the successful management of complex C1/C2 fractures in a patient with concomitant three-vessel CAD.

## Case presentation

**Figure 1:**
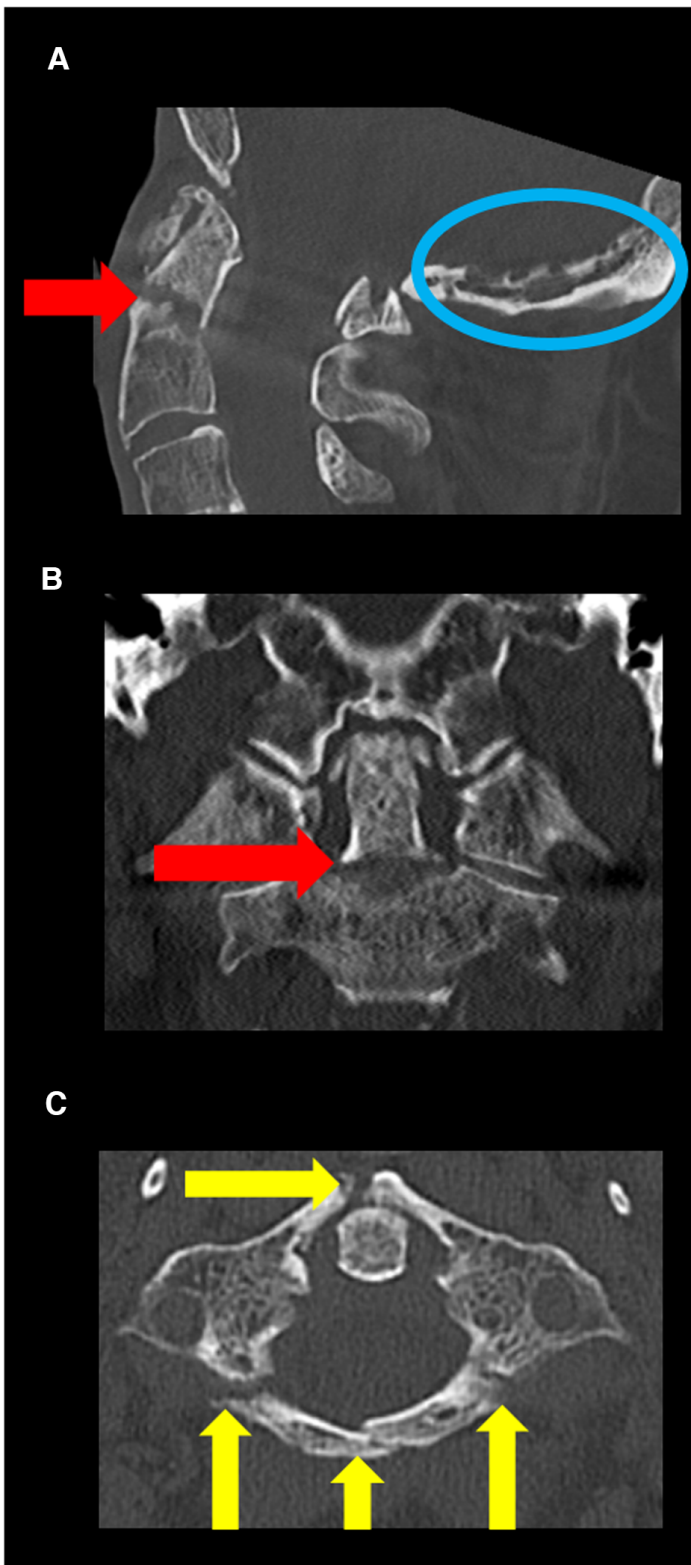
Preoperative CT scan (bone window) of craniocervical junction. (**A**) Sagittal CT revealed a dislocated odontoid type II-C fracture (red arrow) and an intradiploic arachnoid cyst in posterior wall of the posterior fossa (blue circle). (**B**) Coronal CT revealed no lateral dislocation of the fracture of the odontoid process (red arrow). (**C**) Axial CT scan revealed an unstable Jefferson's fracture (with fracture of the anterior arch on the right, dual fractures of the posterior arch and fracture of the left lateral mass of C1) (yellow arrows).

**Figure 2:**
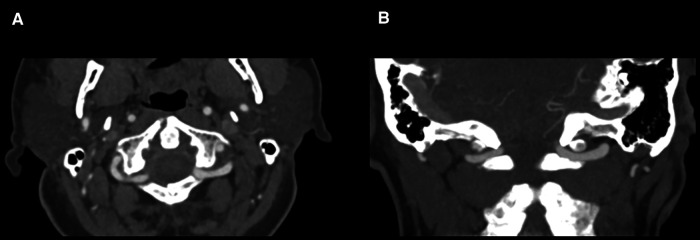
CT angiography of the brain-supplying vessels showing no vascular injury. (**A**) Axial CT scan revealed regular enhancement of internal carotid arteries and V3 segment of vertebral arteries. (**B**) Coronal CT scan revealed hypoplastic intradural segment of right vertebral artery.

A 70-year-old male patient presented in our emergency outpatient clinic after a syncope, causing a bicycle accident. On admission, the patient had no neurological deficits or heart-related complaints. Identified cardiovascular risk factors were hypertension, obesity, smoking and diabetes mellitus. A computed tomography (CT) scan of the cervical spine revealed a displaced odontoid type II-C fracture (17, 18) and an unstable Gehweiler (19) type III injury (Jefferson's fracture) with a fracture of the anterior arch on the right, dual fractures of the posterior arch, and a fracture of the left lateral mass of C1 (Figure 1). A coincident finding was an intradiploic arachnoid cyst in the posterior wall of the posterior fossa (Figure 1). Other lesions included minor head trauma, a fracture of the T2 vertebral body, and rib fractures. CT angiography of cervical spine revealed no vascular injury and regular V3 segment of vertebral arteries (Figure 2). Furthermore, cardiac diagnostics revealed atrial fibrillation and non-ST-segment elevation MI. Coronary angiography confirmed a three-vessel CAD with high-grade stenosis not suitable for coronary stent insertion of left anterior descending artery, ramus circumflex, ramus diagonalis proximalis (RD1) and right coronary artery. Indication for upper cervical spine surgery and bypass surgery was given. The patient's case was evaluated by an interdisciplinary team of neurosurgeons, cardiothoracic surgeons and anesthesiologists to develop the most suitable therapeutic concept.

The following three different strategies were discussed:

1. Conservative therapy of the upper cervical spine fractures and secondary bypass surgery after bone fusion.
2. Conservative therapy of the upper cervical spine fractures and simultaneous bypass surgery.
3. Fusion surgery of the upper cervical spine followed by bypass surgery.

The first concept has the advantage that no fusion surgery of the upper cervical spine needs to be performed if conservative treatment is successful. The disadvantage is that bone fusion might not occur, delaying bypass surgery.

The second option also has the advantage that no fusion surgery is required. During the intervention, bypass surgery could be performed using a Mayfield Headrest System to prevent SCI. Immobilization of the upper cervical spine in a neck brace or halo-fixation would not have been possible during surgery because of the necessary sternotomy. A neck brace or halo-fixation must be worn immediately after surgery. These orthoses might exert pressure on the sternotomy wound leading to delayed wound healing. The physical rehabilitation after coronary artery bypass surgery is also limited while wearing an orthosis. Furthermore, there is a residual risk of neurological impairment during surgery requiring intraoperative neuromonitoring (IOM).

The third option has the advantage that bypass surgery can be performed without any risk of SCI because of the dislocation of the fractures. However, there is a higher intraoperative risk of cardiac events during fusion surgery of the upper cervical spine and a myocardial infarction risk in the time between surgeries. Decision making depends mostly on the severity of MI and how near-term bypass surgery must be performed. Advanced anaesthesiological management is also required. In the present case, considering the patient's age and comorbidities, and due to the fact, that bypass surgery was not emergently indicated, a conservative therapy based on immobilization of the cervical spine in a neck brace was tried. In absence of fracture healing after 4 weeks, surgical approaches were again discussed. Aware of the risk of further dislocation and life-threatening SCI, neurosurgical intervention was performed first in interdisciplinary consent. The setup included general anesthesia, single-shot antibiotics and cardiopulmonary monitoring with resuscitation equipment. Cardiothoracic surgeons were on standby. Occipitocervical fusion was discussed, but not possible, due to the suboccipital intradiploic arachnoid cyst causing a cerebrospinal fluid (CSF) leak after exposure to the skull during surgery (Figure 1). This CSF-leak was closed by bone wax. Finally, the C1-C2 fusion was performed by Harms technique (20) with x-ray guidance, spinal neuronavigation (BrainLab AG, Feldkirchen, Germany) and Doppler ultrasound. Bilateral pedicle screws (Symphony, DePuy Synthes, Raynham, MA, USA) fixed the C2 vertebra, while bilateral mass screws (Symphony, DePuy Synthes, Raynham, MA, USA) were positioned in C1. The posterior atlas fragments were removed by laminectomy, and bone granulate was deposited.

The patient was monitored postoperatively in the intensive care unit (ICU) and later transferred to the neurosurgical ward in stable condition. Postoperative CT scan confirmed correct intraosseous screw positioning. Thromboprophylaxis (enoxaparin) was administered, while aspirin was paused pre- and postoperatively to prevent hemorrhage. Intravenous antibiotics (vancomycin, meropenem, clindamycin) were administered and a lumbar drain was placed. In the course, infection parameters decreased and wound healing was regular. The patient was discharged on day 12. Soft cervical collar was recommended for additional 4 weeks. One month later coronary artery bypass surgery (anastomosis of the left mammary artery to RD1 and the left radial artery to posterior interventricular ramus) was performed. Life-long medication with aspirin was resumed. Rivaroxaban was started due to intermittent atrial fibrillation. The patient was discharged to rehabilitation on day 7.

### Follow-up

**Figure 3:**
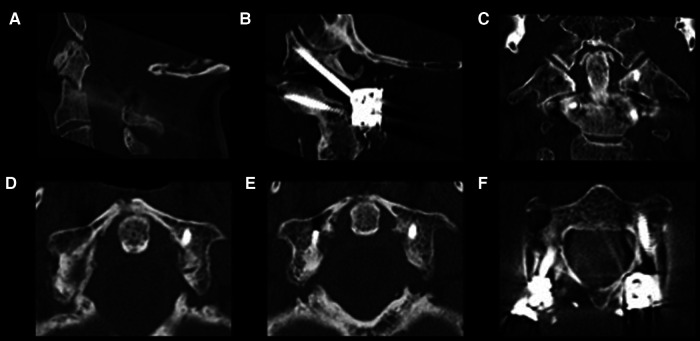
Follow-up sagittal (**A,B**), coronal (**C**) and axial (**D–F**) CT scan revealed proper intraosseous positioning of the screws, furthermore sufficient decompression of atlantoaxial region (**A**) and beginning fusion the odontoid process (**C**) and the anterior arch of C1 (**D,E**).

Three months' follow-up CT scan confirmed beginning fusion of the fractures (fusion of anterior arch of C1 and beginning fusion of odontoid process (Figure 3). The patient did not develop any neurological deficits and recovered completely from both surgeries.

## Discussion

Treating complex C1/C2 fractures with concomitant CAD requiring cardiac surgery is challenging. In addition to surgical indications, the patient's individual risk factors must be considered when determining the definitive therapy (1, 2, 6, 9, 21). Bypass surgery is the standard of care in three-vessel or left main CAD, resulting in lower rates of cardiac or cerebrovascular events compared with percutaneous coronary intervention (22). Preferably, surgery is performed after a stable interval of 2–3 days (14). Myocardial infarction is also a severe complication of noncardiothoracic surgery. For lumbar spine surgery, Harwin et al. (23) reported a significantly higher risk of postoperative MI in men, which increases with age. Furthermore, the incidence was higher in fusion surgeries than in nonfusion surgeries (23). Although systematic evidence is lacking, to best of our knowledge cases of postoperative MI have been described following upper cervical spine fusion (9, 24, 25). In high-risk constellations, it is advisable to provide cardiac and hemodynamic monitoring in the ICU. Decompression of cervical spine trauma at risk for secondary SCI is recommended at least within the first 24 h (21). Cardiovascular complications are a leading cause of death in cervical or high-thoracic (above T6) SCIs because of damage of supraspinal sympathetic nervous system control (26). Consecutive shift towards parasympathetic-dominated heart control leads to bradycardia, orthostatic hypotension, autonomic dysreflexia, and cardiac arrest (26). Hence, early management of cardiac arrhythmias and neurogenic shock is crucial to improve patient outcome.

### Neurosurgical concepts

Fortunately, occipitocervical fusion is rarely required since occipitoatlantal joint injury is seen in only 0,8%–1,1% of extreme trauma (5). In general, therapy for type I injuries and dislocated type II and type III injuries according to Anderson and Montesano (27) is conservative by closed repositioning and rigid immobilization for 6–12 weeks. In case of therapy failure or type III injury with ligamentous instability, C0–C2 fixation with modular occipital plate-rod construct is the strongest way to achieve fusion. On the other hand, the atlantoaxial joint is most prone to instability after cervical spine injury, with occipitocervical fusion reserved for the most complex (28). Atlas fractures account for 2%−13% of cervical spine injuries and occur through axial load (4, 5, 8, 28, 29). Ranging from 30% to 70% of prevalence, C1 fractures are associated with other cervical spine injuries with fractures of the odontoid process being the most significant (8, 28). Combined C1/C2 injuries have a higher rate of neurological deficits, morbidity and mortality. Non-operative management depends on C1-fracture type according to Gehweiler et al. (19): Isolated anterior (type I) and posterior (type II) arch, as well as lateral mass (type IV) and transverse process (type V) fractures are treated by immobilization in a neck brace for 6–12 weeks (8, 28). For type IV fractures with incongruence in the occipitocervical or atlantoaxial joints repositioning followed by halo-fixation for 6–12 weeks is recommended (5). Therapy of Jefferson's fractures (type III) varies depending on the condition of the transverse atlantal ligament (TAL). Conservative treatment by halo-fixation for 12 weeks is possible in stable type III-A fractures. Indication for posterior C1-C2 fusion is due to a large displacement, comminution and unstable Jefferson's fracture (type III-B), characterized by vertical atlantoaxial instability with partial or complete tearing of the TAL (6, 8, 28, 29). Imaging criteria for TAL likely to be disrupted are an overhang of the lateral masses of C1 > 6,9 mm beyond the lateral masses of C2 or enlargement of the anterior atlantodental interval > 3 mm (5, 8). Odontoid fractures account for 9%–15% of cervical spine fractures and are predominantly caused by hyperextension or hyperflexion trauma (8). According to Anderson and D'Alonzo (17), these are classified as type I-III fractures. In general, type I and most type III fractures are treated by external immobilization for 3–6 months (8). Type II fractures represent the most difficulty in management. Grauer et al. (18) proposed a subtype classification for decision-making: Non-displaced type II-A fractures can be treated conservative, while displacement and type II-B fractures require an anterior odontoid screw (8). Type II-C is defined as an unstable, displaced fracture extending from anterior-inferior to posterior-superior. Especially in geriatric patients (Age > 65 years), posterior C1-C2 fusion is the procedure of choice. Nonetheless, pseudarthrosis is seen in conservative therapy of type II odontoid fractures in geriatric patients (5, 6, 9). The literature (Table 1) offers various posterior C1-C2 fusion methods for atlantoaxial instability depending on patient's anatomy, comorbidities and concurrent injuries (8, 42). In general, Harms technique (20) using polyaxial screws is widely accepted as the standard of care (5, 28, 29). Special advantages, disadvantages and risks of different anterior and posterior fixation-techniques are summarized in Table 1. Common risks of all techniques are superficial and deep infections, malpositioning of screws, implant loosening and pseudoarthrosis. Moreover, Alhashash et al. (43) proposed a minimally invasive percutaneous procedure to achieve reduction in surgery time, blood loss and wound-healing disorder. Fusion rates have been reported as high as 100% after posterior atlantoaxial screw fixation (44). Nonetheless, reduced rotational capacity of the cervical spine remains a disadvantage.

**Table 1:** Special advantages, disadvantages and risks of conservative treatment vs. different anterior and posterior fixation techniques for complex C1/C2 fractures.

	Technique	Advantages	Disadvantages and risks
Conservative treatment	Conservative treatment (halo fixation, philadelphia collar)	No surgical risks	Treatment takes many weeks (6–12 weeks) (8, 28), high pseudoarthrosis rate (especially in eldery patients), low compliance
Anterior fixation	Anterior odontoid screw	Preservation of substantial C1-C2 rotatory motion (30), performed in supine position	High complication rate (especially in eldery patients) (31); high pseudoarthrosis rate, dysphagia (30); only treatment of odontoid fracture, in complex C1/C2 fractures (with ligament involvement) “alone” not indicated
	Anterior transarticular fixation	Alternative in case of abberant location of VA, performed in supine position	Inferior resistance of screws compared with posterior fixation (32), VA injury, dypsphagia (33)
	Triple anterior screw fixation (anterior odontoid + transarticular screws)	Performed in supine position	See above: anterior odontoid screw + anterior transarticular fixation
Posterior fixation	Posterior C1-C2 fixation		
	Posterior C1 screw		
	C1 lateral mass screws (through arch C1)	Superior resitance of screws (32), no contact to epidural venous plexus and to C2 nerve root	Risk VA injury, close relation to V3 segment of VA (34), not possible in case of small diamteter of C1 arch
	C1 lateral mass screws (under arch C1)	C1 arch is between the V3 segment of VA and the trajectory (in case of normal variant of VA) (34)	VA injury, especially in case of variants of VA such as persistent first intersegmental artery or fenestration, sacrifice of C2 nerve might be necessary, occipital neuropathic pain syndrome (34–36)
	Posterior C2 screw		
	Pars screws	Short pars screw can be used in case of high-riding VA or medially positioned VA (37)	VA injury
		no difference was seen in the accuracy of placement of C2 pars screws and C2 pedicle screws (38)	
	Pedicle screws	Superior resitance of screws (32), no contact to epidural venous plexus and to C2 nerve root free hand technique, alternative to pedicle screws in case of small pedicles or variants of VA	Risk of injury of VA (but lower than in Magerl technique)(39), up to 20% do not have pedicles large enough to allow for pedicle screws contraindication in case of (hemi-) laminectomy of C2
	Laminar screws		
	Posterior transarticular C1-C2 fixation (Magerl technique)	Less blood loss (compared to Harms technique) (39), lower intrumentation costs (39)	Incidence of VA injury and dural injury highter than in Harms-technique (39), retropharyngeal injury
	(additional) occipital plate	Upper spine completly fixed, high rate of fusion	Risk of CSF fistula (40), risk of sinus injury, no movement of craniocervical junction possible, injury of cerebellar cortex (40) in case of huge enlargement of foramen of magnum placement migth be difficult
	(additional) lateral mass screws of lower segments (C3, C4, C5)	Might be necessary in case of complex fracture of C2	Fracture of lateral mass (41), VA injury, nerve root injury (41)

### C1-C2 fusion: risks and rescue techniques

As mentioned, x-ray guidance, spinal neuronavigation and Doppler ultrasound may be helpful to achieve safer screw insertion. Regarding vascular complications of fusion techniques, the rate of VAI after C1-C2 fusion was found to be 2% per patient and 1% per screw inserted in a systematic review by Ghaith et al. (45) Currier et al. (46) investigated the potential risk of injury to internal carotid artery (ICA) during screw placement into C1. The ICA was found to be at moderate risk on at least one side in 46% of cases and at high risk in 12% of cases (46). The course of these brain-supplying vessels to the atlas, the axis and in the lateral region to the facet joints may differ individually. Therefore, appropriate trajectory should be carefully assessed on CT angiography to reduce injury risk during screw positioning.

Furthermore, ganglionectomy of the C2 nerve root should be avoided, even if the sectioning is generally safe. Superior and inferior mobilization of the C2 nerve root can assist in appropriate exposure for screw insertion and placement of the implants (42).

Ryken et al. (35) concluded that class III medical evidence exists for a variety of treatment options of combined fractures of the atlas and axis in adults, and reviewed different concepts for their management, which included conservative management, posterior C1-C2 fixation, anterior odontoid screw fixation, occipitocervical fusion and posterior transarticular fixation of C1-C2. Furthermore, the anterior transarticular fixation for C1-C2 fractures had been described (also in some cases with additional screw fixation of odontoid process, so called ‘triple anterior screw fixation’) (47–51). Agrillo et Mastronardi (48) described this technique in a case of a 92-year-old man with posterior arch fracture of the atlas, associated with a type II odontoid fracture. The advantage of this procedure is that it can be performed in supine position. For Jefferson’s fracture together with odontoid type II-C fracture Guiot et Fessler (52) described one case which they treated by ‘triple anterior screw fixation’.

However, in case of Jefferson’s fracture a shift of the fragments of C1 might be possible using this stabilization technique. Levine et Edwards (49) mentioned that this technique is fraught “with great surgical risks and are not easily accomplished”. In our opinion in case of fracture of C1 and C2 with three-vessel CAD with high-grade coronary artery stenosis this option should also be discussed in future.

### Nontraumatic intradiploic arachnoid cyst

Weinand et al. (53) first introduced the term intradiploic arachnoid cyst. Nontraumatic intradiploic cysts (the only extradural manifestation of intracranial arachnoid cysts) expand the diploic space through a small congenital dural defect due to CSF-pulsation while the outer table of the skull is intact. In general, it is a coincident radiological finding in asymptomatic patients without trauma history. Nonetheless, cases with symptoms such as headache, dizziness, and local pain have been reported in the literature (54–57). CT scan reveals solitary or multiple, intradiploic, CSF-density, symmetrical well-demarcated lytic lesions with focal cortical erosions (53, 57). Differential diagnoses include epidermoid/dermoid cyst, hemangioma, intraosseus meningioma, eosinophilic granuloma, plasmacytoma, osteogenic sarcoma and metastasis (53, 55–57). Intradiploic arachnoid cysts are primary documented in the occipital bone (53–56), whereas our patient presented one in the suboccipital bone. This cyst localization made occipitocervical fusion impossible. The surgical treatment consists of the excision of the pedicle by craniectomy and repair of dural and skull defects. However, given the benign nature of the lesion, we strongly recommend radiological follow-up for small cysts in asymptomatic patients.

## Conclusion

Complex C1/C2 fractures with concomitant severe CAD remain challenging and require an individual approach to receive upper cervical spine stability and to prevent both, SCI and cardiovascular complications. If the injuries are unstable and bypass surgery is not immediately necessary, surgical intervention is indicated. Therefore, interdisciplinary cooperation is essential to determine optimal therapeutic algorithms.

## Data Availability

The original contributions presented in the study are included in the article, further inquiries can be directed to the corresponding author.
